# Non-invasive assessment of fluid responsiveness by changes in partial end-tidal CO_2 _pressure during a passive leg-raising maneuver

**DOI:** 10.1186/2110-5820-2-9

**Published:** 2012-03-26

**Authors:** Manuel Ignacio Monge García, Anselmo Gil Cano, Manuel Gracia Romero, Rocío Monterroso Pintado, Virginia Pérez Madueño, Juan Carlos Díaz Monrové

**Affiliations:** 1 Unidad de Investigación Experimental. Servicio de Cuidados Críticos y Urgencias. Hospital SAS de Jerez, Jerez de la Frontera, C/Circunvalación s/n, 11407 Jerez de la Frontera, Spain

**Keywords:** Fluid responsiveness, Passive leg raising, Cardiac output, Preload, Esophageal Doppler, Partial end-tidal CO_2_

## Abstract

**Background:**

The passive leg-raising (PLR) maneuver provides a dynamic assessment of fluid responsiveness inducing a reversible increase in cardiac preload. Since its effects are sudden and transitory, a continuous cardiac output (CO) monitoring is required to appropriately assess the hemodynamic response of PLR. On the other hand, changes in partial end-tidal CO_2 _pressure (PETCO_2_) have been demonstrated to be tightly correlated with changes in CO during constant ventilation and stable tissue CO_2 _production (VCO_2_). In this study we tested the hypothesis that, assuming a constant VCO_2 _and under fixed ventilation, PETCO_2 _can track changes in CO induced by PLR and can be used to predict fluid responsiveness.

**Methods:**

Thirty-seven mechanically ventilated patients with acute circulatory failure were monitored with the CardioQ-ODM esophageal Doppler. A 2-minutes PLR maneuver was performed. Fluid responsiveness was defined according to CO increase (responders ≥ 15%) after volume expansion.

**Results:**

PLR-induced increases in CO and PETCO_2 _were strongly correlated (R^2 ^= 0.79; *P *< 0.0001). The areas under the receiver-operating characteristics (ROC) curve for a PLR-induced increase in CO and PETCO_2 _(0.97 ± 0.03 SE; CI 95%: 0.85 to 0.99 and 0.94 ± 0.04 SE; CI 95%: 0.82 to 0.99; respectively) were not significantly different. An increase ≥ 5% in PETCO_2 _or ≥ 12% in CO during PLR predicted fluid responsiveness with a sensitivity of 90.5% (95% CI: 69.9 to 98.8%) and 95.2% (95% CI: 76.2 to 99.9%), respectively, and a specificity of 93.7% (95% CI: 69.8 to 99.8%).

**Conclusion:**

Induced changes in PETCO_2 _during a PLR maneuver could be used to track changes in CO for prediction of fluid responsiveness in mechanically ventilated patients with acute circulatory failure, under fixed minute ventilation and assuming a constant tissue CO_2 _production.

## Background

The passive leg-raising (PLR) maneuver provides a dynamic assessment of preload dependence inducing a transient and reversible increase in cardiac preload. The abrupt transfer of blood contained in the venous reservoir of the legs and splanchnic compartment while moving the patient from a semirecumbent to supine position with legs elevated increases cardiac preload as a 'self-volume challenge' and, when both ventricles are operating in the steep part of the Frank-Starling curve, also improves cardiac output (CO) [1]. This maneuver has been demonstrated to predict fluid responsiveness in many studies over a wide population, including clinical situations in which other parameters of fluid responsiveness have failed, such as patients with cardiac arrhythmias or with spontaneous breathing [1-3]. However, since the hemodynamic effects of PLR are usually sudden and transient, a fast-response continuous CO monitor is required to detect these changes and to characterize fluid responder patients accurately [4-6]. In this regard, several monitoring techniques have been proposed for this purpose: echocardiography [2,3,7], arterial pulse contour analysis [8,9], bioreactance [10], esophageal Doppler [1,11,12], and so on. Nevertheless, the need for measuring CO usually limits the widespread application of this test to a specific group of patients requiring expensive, burdensome or invasive hemodynamic monitoring systems.

The relationship between CO and partial end-tidal CO_2 _pressure (PETCO_2_) has been known for decades [13,14], so measurement of PETCO_2 _has been proposed to confirm the restoration of spontaneous circulation in patients with cardiac arrest [15], but also as a quantitative indicator of hemodynamic effectiveness of precordial compression during cardiopulmonary resuscitation [16]. Moreover, since PETCO_2 _is mainly determined by tissue CO_2 _production (VCO_2_), alveolar ventilation and CO (that is, pulmonary blood flow) [17], when stable metabolic conditions are assumed and minute ventilation is kept constant, acute changes in PETCO_2 _have been shown to correlate strongly with changes in CO in experimental [18-24] and clinical [25,26] settings. Thus, PETCO_2 _has been suggested as a noninvasive alternative for continuous assessment of CO in different shock states [20].

The aim of the present study, therefore, was to assess whether PETCO_2 _monitoring can track changes in CO induced by PLR and can be used as a noninvasive surrogate for predicting fluid responsiveness in mechanically ventilated patients with acute circulatory failure requiring fluid administration.

## Methods

This study was prospectively conducted in a 17-bed adult multidisciplinary Intensive Care Unit of the Hospital del SAS Jerez de la Frontera. The study protocol was approved by the Institutional Ethics Committee of the Hospital Jerez of the Andalusian Health Service and endorsed by the Scientific Committee of the Spanish Society of Intensive Care, Critical and Coronary Units (SEMICYUC). Written informed consent was obtained from each patient's next of kin.

### Patients

Inclusion criteria were patients with controlled mechanical ventilation and for whom the attending physician decided to give fluids due to the presence of at least one of the following signs of circulatory failure: systolic blood pressure ≤ 90 mmHg or a decrease ≥ 50 mmHg in a previously hypertensive patient; the need of vasopressor drugs; urine output ≤ 0.5 ml/kg/hr during at least two hours; heart rate > 100 beats/minute; and presence of skin mottling or delayed capillary refilling. Exclusion criteria were age < 18 years, pregnancy, any contraindication for the use of esophageal Doppler (recent esophageal surgery, malformation, varicose or tumor) or to perform PLR (intracranial hypertension, deep venous thrombosis, the use of venous elastic compression stockings, or limb and pelvic fractures).

To avoid changes in alveolar ventilation, patients were ventilated in volume control mode and spontaneous respiratory movements were temporally suppressed with a neuromuscular blockade agent (0.1 mg/kg vecuronium bromide) if detected in airway pressure trace in the respiratory monitor.

### Measurements

#### Respiratory measurements

PETCO_2 _was continuously measured at the tip of the endotracheal tube using a sidestream infrared gas analyzer (compact airway module M-COVX, Datex-Ohmeda, Helsinki, Finland) integrated into the patient monitor (S/5, Datex-Ohmeda, Helsinki, Finland) and recorded online in a laptop computer every 10 seconds using proprietary data acquisition software (S/5 Collect software, version 4.0; Datex-Ohmeda, Helsinki, Finland). The validation of this compact modular metabolic monitor for use in mechanically ventilated patients in the ICU has been published elsewhere [27]. The precision of the gas analyzer module for PETCO_2 _measurements was calculated as twice its coefficient of variation (CV = SD/mean), determined in all studied patients at baseline during stable hemodynamic and respiratory conditions. The least significant change (LSC) was calculated as the precision × √2, which sets the minimum percentage change between successive measurements that can be considered not due to random error and therefore representing a real change in PETCO_2 _[28]. Since as the random error increases, larger effects are necessary to detect a real change, responsiveness of the gas analyzer for PETCO_2 _measurements was also calculated, which refers to the ability to detect a change over time, providing an estimation of the 'sensitivity to change' of a measurement tool [29]. We calculated the Guyatt's Responsiveness Index (GRI) as GRI = LSC divided by the standard deviation of the PLR-induced changes in PETCO_2_. A GRI value ≥ 0.8 is considered a high responsiveness [30].

#### Hemodynamic measurements

Hemodynamic monitoring was performed using the CardioQ-ODM™ esophageal Doppler monitor (Deltex Medical, Chicester, UK). This device allows minimally invasive real-time CO monitoring by measuring aortic blood flow velocity at the descending thoracic aorta, assuming a constant aortic diameter (obtained from a nomogram based on the patient's age, weight and height) and that a constant proportion of the CO flows through the descending aorta [31]. This monitor also provides other flow-related hemodynamic parameters: the corrected flow time (FTc), which is adjusted to the heart rate using Bazett's formula (FTc = the systolic blood flow time divided by the square root of the cycle time) and has been proposed as a gross estimation of the loading condition of the heart [32]; and the mean acceleration (Acc), which represents the mean acceleration of the aortic blood flow and has been suggested as an indicator of the left ventricular systolic function [33]. The Doppler probe was inserted into the esophagus via the nasal route and advanced until it achieved the maximal aortic blood flow velocity signal. The gain setting was then adjusted to obtain the optimum outline of the aortic velocity waveform. Hemodynamic parameters were continuously recorded every 10 seconds for further offline analysis.

All Doppler measurements were performed by the same observer (MIMG). The intraobserver reproducibility for CO measurements was determined using the Bland-Altman test analysis in ten randomly selected patients over a one-minute period and described as bias ± limits of agreements (1.96SD), and presented as percentages and absolute values (in brackets). We also calculated precision and LSC for Doppler CO measurements at pre-infusion and post-infusion stages. Since Doppler parameters were averaged during one minute before and after fluid administration, precision was determined by the coefficient of error (CE = CV/√N, where N = 6 is the number of measurements) and LSC as 2√2 × CE [28].

### Study protocol

**Figure 1:**
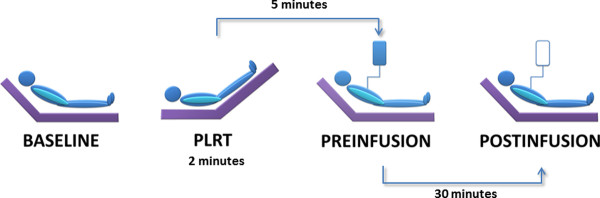
**Study protocol**.

The study protocol was performed in four sequential stages (Figure 1). A first set of measurements was recorded with the patient in the semirecumbent position (baseline). Next the PLR maneuver was performed by setting the patient in the supine position and simultaneously raising the patient's leg to 45° to maximize the hemodynamic effect of the test (PLR) [9]. The patient was then returned to the semirecumbent position and, after five minutes, a new set of measurements was obtained before (pre-infusion) and immediately after fluid administration (post-infusion), which consisted of 500 ml of a synthetic colloid (Voluven 6% hydroxyethyl starch solution 130/0.4; Fresenius Kabi, Bad Homburg, Germany) administered over 30 minutes. The hemodynamic and PETCO_2 _measurements during each protocol period were expressed as an average of the measurements collected during one minute, except for the PLR stage, in which they were recorded at the moment at which they reached their maximum values [1].

Supportive therapies, ventilatory settings and vasopressor therapy were kept unchanged throughout the study period.

### Statistical analysis

Normal distribution of data was tested using the D'Agostino-Pearson test. All variables except PETCO_2 _were normally distributed. The results are expressed as means ± SD or as median and the interquartile range (IQR), as appropriate. Sample size was calculated for detection of differences of 0.10 with known expected area under the ROC curve [34]. We selected a type I error of 0.05 and a type II error of 0.2, assuming that fluid responsiveness occurs in 50% of ICU patients [35]. Patients were classified according to CO increase after fluid administration as responders (≥ 15%) and non-responders, respectively [1,11]. To validate this cutoff we determined that every responder patient increased CO above the individual LSC value [28]. Differences between responders and non-responders were compared by means of an independent sample *t*-test for hemodynamic parameters and by the Mann-Whitney *U *test for PETCO_2 _measurements. The effects of fluid administration on hemodynamic parameters were assessed using a paired Student's *t*-test and by Wilcoxon rank sum test for PETCO_2 _measurements. The relationships between variables were analyzed using a linear regression method. The ability of PETCO_2 _to track directional changes in CO (trending capability) during PLR and fluid administration was tested using a concordance analysis. Concordance was defined as the percentage of data in which the direction of change was in agreement [36]. The area under the ROC curves for PLR-induced changes in CO (ΔCO_PLR_) and PETCO_2 _(ΔPETCO_2-PLR_), and pre-infusion FTc according to volume expansion (VE) response were calculated and compared using the Hanley-McNeil test. ROC curves are presented as area ± SE (95% confidence interval). A *P *value < 0.05 was considered statistically significant. All statistical analyses were two-tailed and were performed using MedCalc software for Windows version 11.6.1.0 (MedCalc Software bvba, Mariakerke, Belgium).

## Results

### Patients' characteristics

From January to May 2011, 37 consecutive patients were included. Two patients were excluded because of inability to acquire an adequate Doppler signal. Patients' characteristics are summarized in Table 1, including 21 fluid responders (57%) and 16 non-responders. All the patients had sinus rhythm, although transient supraventricular extrasystoles were observed during the study in some patients (13%).

**Table 1:** Characteristics and demographic data of study population

*Age (years)*	64 ± 13
*Gender (M/F)*	16/21
*Weight (kg)*	77 ± 17.2
*Height (cm)*	166.9 ± 7.6
*Body surface area (m^2^)*	1.88 ± 0.21
*Body mass index (Kg m^-2^)*	25.9 (23.4 to 31.2)
*APACHE II score at admission*	18.8 ± 7.3
*ICU survival rate (%)*	24 (65%)
*Plasma lactate level, mmol/L*	2.79 ± 1.82
*Ventilator settings*	
*Tidal volume, mL/Kg predicted body weight*	8.1 ± 1.2
*Respiratory rate, b.p.m*.	20 (18 to 20)
*Total PEEP, cm H_2_O*	6.4 (4 to 8)
*Mean airway pressure, cmH_2_O*	12 (9.4 to 13)
*Peak airway pressure, cmH_2_O*	31.1 ± 6.2
*FiO_2_,%*	0.73 ± 0.2
*Vasoactive agents, n; dose (μg kg^-1 ^min^-1^)*	
*Norepinephrine*	22; 0.23 (0.08 to 0.33)
*Dobutamine*	6; 6.2 (3.3 to 9.5)
*Analgesia and sedative drugs*	
*Fentanyl, n; dose (μg kg^-1 ^h^-1^)*	24; 1.58 ± 0.53
*Remifentanyl, n; dose (μg kg^-1 ^min^-1^)*	13; 0.14 ± 0.04
*Midazolam, n; dose (mg kg^-1 ^h^-1^)*	24; 0.09 ± 0.03
*Propofol, n; dose (mg kg^-1 ^min^-1^)*	2; 0.79 (0.7 to 0.89)
*ARDS, n*	3
*Days on mechanical ventilation*	1 (1 to 1)
*ICU length of stay, days*	8 (5 to 16)
*Reason for fluid administration, n (%)*	
*Hypotension*	11 (30%)
*Oliguria*	30 (81%)
*Decrease vasoactive dosage*	19 (51%)
*Sepsis/Septic shock*	26 (70%)
*Abdominal*	11 (30%)
*Pulmonary*	10 (27%)
*Urological*	2 (7%)
*Neurological*	2 (7%)
*Other*	1 (3%)

APACHE, Acute Physiologic and Chronic Health Evaluation; ARDS, acute respiratory distress syndrome; F, female; FiO_2_, inspired oxygen fraction; ICU, intensive care unit; M, male; PEEP, positive end-expiratory pressure; SaO_2_, arterial oxygen saturation. Values are expressed as mean ± SD, median (25^th ^to 75^th ^percentile) or absolute numbers, as appropriate

The intraobserver variability for esophageal Doppler CO measurements was 0.6 ± 4.9% (-0.02 ± 0.21 L/min). At pre-infusion, the precision and mean LSC for Doppler CO measurements were 2.3% (IQR: 1.7 to 3%; 95% CI: 1.8 to 2.7%) and 3.2% (IQR: 2.4 to 4.2%; 95% IC: 2.5 to 3.8%), respectively; at post-infusion, these values were 1.9% (IQR: 1.3 to 3.9%; 95% CI: 1.5 to 2.6%) and 2.6% (IQR: 1.8 to 5.4%; 95% IC: 2.1 to 3.6%). For PETCO_2 _measurements, the precision and mean LSC were 1.3% (IQR: 1.04 to 1.7%; 95% CI: 1.09 to 1.57%) and 1.84% (IQR: 1.47 to 2.41%; 95% CI: 1.54 to 2.22%), respectively. The responsiveness of the gas analyzer according to Guyatt's Responsiveness Index was 1.58 (IQR: 1.26 to 2.07; 95% IC: 1.32 to 1.91).

### Effects of passive leg-raising maneuver

Overall, the PLR maneuver increased CO by 15.9 ± 9.1% (95% CI: 12.9 to 18.9%; *P *< 0.0001), stroke volume (SV) by 15.5 ± 10.2% (95% CI: 12.1 to 18.9%; *P *< 0.0001), FTc by 8.4 ± 6% (95% CI: 6.4 to 10.4%; *P *< 0.0001), mean arterial pressure (MAP) by 5.4% (IQR: 1.3 to 10.2%; 95% CI: 1.8 to 7.6%; *P *< 0.0001), arterial pulse pressure (PP) by 10.1% (IQR: 2.3 to 21.8; *P *< 0.0001), and PETCO_2 _by 5.32 ± 3.09% (95% CI: 4.29 to 6.35%; *P *< 0.0001). Neither heart rate nor Acc changed during PLR.

In fluid-responder patients, ΔPETCO_2-PLR _was 7.34 ± 2.26% (95% CI: 6.32 to 8.37%) with an absolute increase of 2.54 ± 0.99 mmHg (95% CI: 2.09 to 2.99 mmHg), whereas in non-responders, this value was 2.66 ± 1.69% (95% CI: 1.76 to 3.56%), with an absolute increase of 0.92 ± 0.61 mmHg (95% CI: 0.59 to 1.24 mmHg). All volume responders showed a ΔPETCO_2-PLR _greater than their individual baseline LSCs.

**Figure 2:**
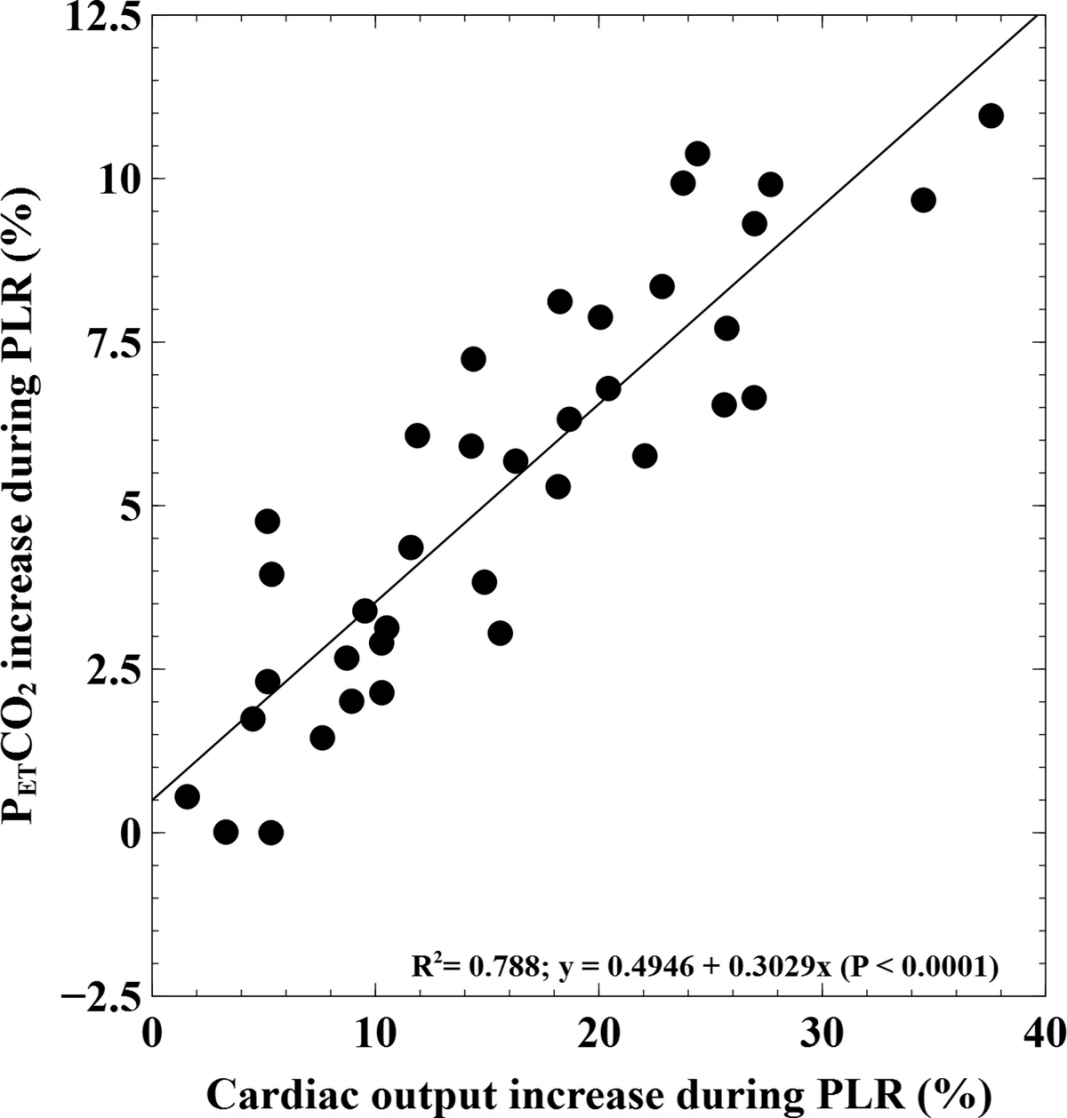
**Linear regression analysis of the relationship between PLR-induced changes in cardiac output and PETCO_2_**. PETCO_2_, partial end-tidal CO_2_ pressure; PLR, passive leg raising.

The CO increase during PLR strongly correlated with ΔPETCO_2-PLR _(R^2 ^= 0.79; *P *< 0.0001) but was weaker with PLR-induced changes in arterial PP (R^2 ^= 0.29; *P *< 0.001) (Figure 2). The directional changes between ΔPETCO_2-PLR _and CO induced by PLR displayed a 97% concordance.

After the PLR maneuver, at the pre-infusion period, all the parameters returned to similar baseline values.

### Effects of volume expansion

In the entire study population, VE increased CO by 18.9 ± 17.1% (95% CI: 13.1 to 24.6%; *P *< 0.0001), SV by 17.4% (IQR: 6.1 to 31.2%; 95% CI: 9.3 to 22.3; *P *< 0.0001); FTc by 8.4 ± 8.7% (95% CI: 5.5 to 11.3%; *P *< 0.0001), MAP by 4.7% (IQR: 1.2 to 13.4%; 95% CI: 2.3 to 10.6%; *P *= 0.0001); arterial PP by 12.5% (IQR: 1.6 to 25.9; *P *< 0.001); and PETCO_2 _by 1.69 ± 3.55% (95% CI: 0.51 to 2.88% mmHg; *P *< 0.01). All the VE-responder patients exhibited an increase in CO after VE greater than their individual LSC values for Doppler CO measurements.

**Figure 3:**
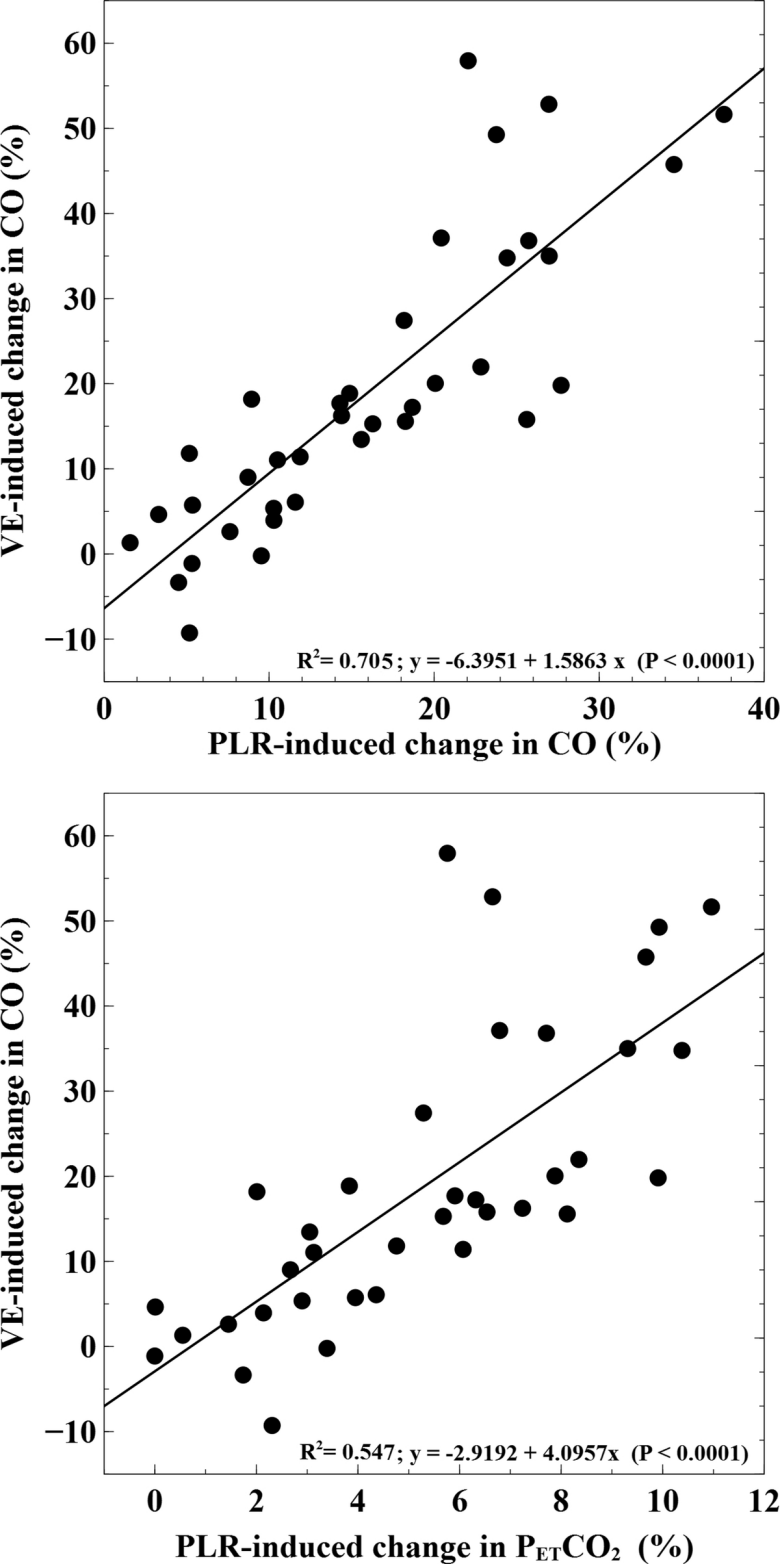
**Linear regression analysis of the relationship between cardiac output changes induced by volume expansion and PLR-induced changes in cardiac output and PETCO_2_**. CO, cardiac output; PLR, passive leg raising; VE, volume expansion.

The VE-induced increase in CO was correlated with changes in PETCO_2_, PP and FTc after fluid administration (R^2 ^= 0.56, R^2 ^= 0.45 and R^2 ^= 0.7, respectively; *P *< 0.0001). Changes between VE-induced increases in PETCO_2 _and CO showed a 59% concordance. Furthermore, CO increase after fluid administration was also correlated with ΔCO_PLR _and ΔPETCO_2-PLR _(R^2 ^= 0.7 and R^2 ^= 0.55, respectively; *P *< 0.0001) (Figure 3). So, the greater the changes in CO and PETCO_2 _during the PLR maneuver, the greater the expected increase in CO after VE. No correlation between pre-infusion FTc values and VE-induced changes in CO was observed (R^2 ^= 0.09; *P*: n.s.).

The hemodynamic and PETCO_2 _measurement throughout the four stages are summarized in Table 2.

**Table 2:** Effects of passive leg raising maneuver and volume expansion on hemodynamics and partial end-tidal CO_2_

	Baseline	PLR	Preinfusion	After VE
**CO, L/min**				
*Responders*	5.40 ± 2.25	6.54 ± 2.53^b^	5.33 ± 2.43	6.81 ± 2.81^c^
*Non-responders*	6.51 ± 2.47	7.03 ± 2.73^b^	6.48 ± 2.52	6.85 ± 2.89^c^
**SV, mL**				
*Responders*	53.5 ± 26.3^a^	63.8 ± 26.5^b^	52.5 ± 26.9^a^	68.1 ± 27.5^c^
*Non-responders*	76.4 ± 31.3	82.7 ± 33.5^b^	76.3 ± 30.9	81.5 ± 33.1^c^
**HR, b.p.m**.				
*Responders*	106 ± 25^a^	106 ± 24^a^	106 ± 26^a^	103 ± 24^a^
*Non-responders*	88 ± 20	87 ± 18	87 ± 20	86 ± 19
**FTc, ms**				
*Responders*	292 ± 51^a^	323 ± 50^b^	291 ± 52^a^	327 ± 54^c^
*Non-responders*	343 ± 61	360 ± 74^b^	342 ± 62	355 ± 83
**Acc, m/s**^**2**^				
*Responders*	9.6 ± 4.5	9.7 ± 4.9	9.3 ± 4.6	10.4 ± 5.4^c^
*Non-responders*	10.3 ± 3.9	10.5 ± 4.1	10.3 ± 3.9	10.7 ± 4.7
**SAP, mmHg**				
*Responders*	107 ± 21	120 ± 21^b^	108 ± 21	122 ± 25^c^
*Non-responders*	107 ± 14	111 ± 16^b^	105 ± 15	111 ± 18^c^
**DAP, mmHg**				
*Responders*	61 ± 11	65 ± 12^b^	61 ± 12	64 ± 12^c^
*Non-responders*	63 ± 11	66 ± 10^b^	63 ± 10	65 ± 10^c^
**MAP, mmHg**				
*Responders*	76 ± 14	83 ± 13^b^	75 ± 14	83 ± 15^c^
*Non-responders*	78 ± 12	82 ± 12^b^	77 ± 12	81 ± 13^c^
**PP, mmHg**				
*Responders*	45.8 ± 15.7	55.3 ± 18.3^b^	46.9 ± 13.6	57.7 ± 19.7^a, c^
*Non-responders*	42.1 ± 10.1	45.2 ± 11.9^b^	42.1 ± 11.3	45.9 ± 12.9^c^
**CPO, W**				
*Responders*	0.92 ± 0.44	1.22 ± 0.49^b^	0.91 ± 0.47	1.25 ± 0.51^c^
*Non-responders*	1.10 ± 0.38	1.26 ± 0.46^b^	1.11 ± 0.43	1.23 ± 0.53^c^
**TSVR, dyn·s·cm**^**-5**^				
*Responders*	1264 ± 442	1143 ± 368^b^	1299 ± 481	1130 ± 451^c^
*Non-responders*	1124 ± 535	1091 ± 510	1119 ± 504	1133 ± 538
**PETCO**_2_**, mmHg**				
*Responders*	35 (30 to 37)	38 (33 to 40)^b^	35 (29 to 37)	36 (30 to 38)^c^
*Non-responders*	34 (30 to 37)	35 (30 to 38)^b^	34 (29 to 37)	34 (28 to 37)

Acc, mean aortic blood flow acceleration; CO, cardiac output; CPO, cardiac power output (mean arterial pressure x cardiac output/451);, DAP, diastolic arterial pressure; HR, heart rate; FTc, aortic blood flow corrected time; MAP, mean arterial pressure; PETCO_2_, partial end-tidal CO_2 _pressure; PP, arterial pulse pressure (systolic minus diastolic pressure); SAP, systolic arterial pressure; SV, stroke volume; TSVR, total systemic vascular resistance

^a^P < 0.05 versus non-responders; ^b^P < 0.05 versus baseline; ^c^P < 0.05 versus preinfusion. Data are expressed as mean ± SD, except for PETCO_2 _as median (25^th ^to 75^th ^percentiles)

### Prediction of fluid responsiveness

**Figure 4:**
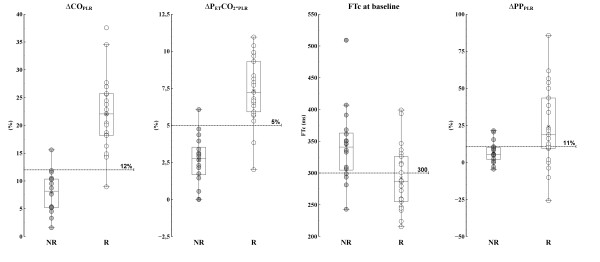
**Individual values and box-plot of studied fluid-responsiveness parameter in responders (open circles) and non-responders (closed circles)**. ΔCO_PLR_, cardiac output changes induced by passive leg raising (PLR); ΔPETCO_2-PLR _= PETCO_2 _changes induced by PLR; ΔPP_-PLR_, arterial pulse pressure changes induced by PLR; FTc: corrected flow time at pre-infusion time.

**Figure 5:**
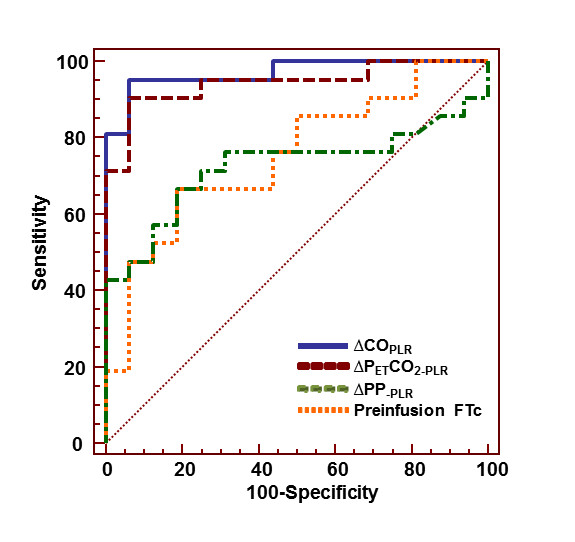
**Comparison of receiver operating characteristics curves regarding the ability of studied fluid responsiveness parameters to discriminate responder patients (cardiac output increase ≥ 15%) and nonresponder patients after volume expansion**. ΔCO_PLR_, cardiac output changes induced by passive leg raising (PLR); ΔPETCO_2-PLR_, PETCO_2 _changes induced by PLR; ΔPP_-PLR_, arterial pulse pressure changes induced by PLR; FTc: corrected flow time at pre-infusion time.

The area under the ROC curve for ΔCO_PLR _(0.97 ± 0.03; 95% CI: 0.85 to 0.99) and ΔPETCO_2-PLR _(0.94 ± 0.03; 95% CI: 0.82 to 0.99) was not significantly different but was higher than for pre-infusion FTc (0.75 ± 0.08; 95% CI: 0.58 to 0.88; *P *< 0.01) and baseline PP (0.73 ± 0.09; 95% CI: 0.55 to 0.86; *P *< 0.01) (Figures 4 and 5). An absolute increase in PETCO_2 _value ≥ 2 mmHg during PLR was associated with a positive response to fluid administration in all cases.

The comparison of predictive performance of different hemodynamic parameters and ΔPETCO_2-PLR _for detecting fluid responsiveness is given in Table 3.

**Table 3:** Predictive performance for detecting fluid responsiveness

	Threshold	Sensitivity	Specificity	PV +	PV -	LR +	LR -
**ΔCO**_**PLR**_	**12%**	**95.2%**	**93.7%**	**95.2%**	**93.7%**	**15.24**	**.05**
**[95% CI]**		[76.2-99.9%]	[69.8-99.8%]	[75.5-99.9%]	[69.8-99.8%]	[13-17.9]	[0.003-0.8]
**ΔPETCO**_**2-PLR**_	**5%**	**90.5%**	**93.7%**	**95%**	**88.2%**	**14.48**	**.1**
**[95% CI]**		[69.6-98.8%]	[69.8-99.8%]	[75.1-99.9%]	[62.6-98.7%]	[12-17.5]	[0.01-1]
**FTc**	**300 ms**	**66.7%**	**81.2%**	**82.4%**	**65%**	**3.56**	**.41**
**[95% CI]**		[43-85.4%]	[54.4-96%]	[55.6-96.5%]	[40.8-84.6%]	[2.4-5.2]	[0.1-1.3]
**ΔPP**_**PLR**_	**11%**	**66.7%**	**81.2%**	**82.4%**	**65%**	**3.56**	**.41**
**[95% CI]**		[43-85.4%]	[54.4-96%]	[55.6-96.5%]	[40.8-84.6%]	[2.4-5.2]	[0.1-1.3]

ΔCO_PLR_, passive leg raising induced-change in cardiac output; ΔPETCO_2-PLR_, passive leg raising-induced change in partial end-tidal CO_2_;, ΔPP_-PLR_, passive leg raising-induced change in arterial pulse pressure; FTc, aortic blood flow corrected time; PV+, positive predictive value; PV -, negative predictive value; LR+, positive likelihood ratio; LR-, negative likelihood ratio

## Discussion

The main finding of this study is that, under fixed minute ventilation and assuming a constant tissue CO_2 _production, acute changes in partial end-tidal CO_2 _pressure during the passive leg-raising maneuver are strongly correlated with changes in cardiac output measured by an esophageal Doppler and could provide a reliable and non-invasive method for predicting fluid responsiveness in mechanically ventilated patients with acute circulatory failure.

Although intravascular volume therapy remains a keystone in the treatment of patients with acute circulatory failure, the benefits of fluid administration seem to be defined within relatively narrow boundaries: on the one hand, excessive fluid therapy appears to be associated with higher morbimortality in critical ill patients and, on the other hand, inadequate fluid resuscitation could aggravate tissue hypoperfusion and organ dysfunction [37]. Furthermore, for physiological reasons, the hemodynamic response to a fluid challenge is not easily predictable, since the expected improvement in CO is only observed in half the patients [35]. In this regard, the PLR maneuver has been proposed as a reliable method for predicting fluid responsiveness, challenging the cardiovascular system to a reversible and transient increase in cardiac preload by mobilizing venous blood contained in the lower limbs and abdominal compartment toward the intrathoracic vessels. Only if both ventricles are operating in the steep part of the cardiac function curve will this increment in preload result in a significant increase in CO and, therefore, in a similar response after volume administration [6].

Our results confirm the ability of the PLR maneuver to detect preload dependence and the strong relationship between changes induced by PLR and VE-induced increases in CO [6,34]. Moreover, the ΔCO_PLR _cutoff for predicting fluid responsiveness is also consistent with previous studies in which esophageal Doppler was employed [1,11] and similar to the recommended threshold value for interpretation of PLR maneuver effects [6,34]. Accordingly, when CO increased above 12%, 95% of the studied patients increased their CO after fluid administration.

Exhaled or end-tidal CO_2 _is mainly determined by pulmonary blood flow (that is, CO), metabolic CO_2 _production (VCO_2_) and alveolar ventilation; so PETCO_2 _varies directly with VCO_2 _and CO and inversely with alveolar ventilation [17]. Therefore, assuming that during PLR the systemic metabolic rate remained relatively constant (and minute ventilation was kept unchanged), then changes in PETCO_2 _should predominantly reflect variations in pulmonary blood flow and thus, indirectly, changes in CO. The strong relationship observed between changes in PETCO_2 _and CO during PLR supports this hypothesis and it is also in agreement with numerous studies performed under similar conditions [18-26]. For this reason, the measurement of PETCO_2 _has been advocated as a simple and noninvasive parameter of the circulatory status for monitoring the resumption of spontaneous circulation and the efficacy of chest compressions during cardiac arrest [15] but also as a prognostic indicator in cardiopulmonary resuscitation [38] and emergency trauma surgery [39].

According to previous observations [22,25], the increase in PETCO_2 _induced by PLR could be explained at least by two mechanisms. First, if PLR increases venous return and pulmonary blood flow, then CO_2 _delivery to the lungs (that is, pulmonary arterial CO_2 _content) and CO_2 _removal should be increased. Second, by increasing pulmonary perfusion pressure, PLR could recruit previous collapsed pulmonary vessels and reduce alveolar spaces with high ventilation-to-perfusion mismatch or alveolar dead space. This latter phenomenon could be particularly striking in patients with severely impaired pulmonary perfusion pressure, as seen in deep hemorrhagic shock [18,19,21,23] or patients with alveolar overdistension due to high levels of PEEP [40]. In this latter regard, a recent study by Fougéres *et al. *[41] demonstrated that in patients with ARDS who were ventilated with high levels of PEEP, the PLR maneuver improved the cardiac index but also was associated with a decrease in pulmonary vascular resistance, which suggested that by increasing central blood volume, PLR decreased PEEP-induced West zones 1 with subsequent decrease in alveolar dead space. Unfortunately, since we did not measure arterial CO_2 _pressure, we cannot estimate the relative influence of changes in dead space during PLR. In any case, according to the above explanation, whatever the underlying mechanism, the increase in PETCO_2 _is determined ultimately to an effective increase in pulmonary blood flow and improved efficiency of alveolar CO_2 _excretion [22,25].

Although the relationship between CO and PETCO_2 _was initially described as linear [14,22,24,25], other authors have suggested that a logarithmic function better defines this association [18,20,21]. According to the latter, in low-flow states (as during cardiac arrest or severe hemorrhagic shock), for a given change in CO a larger variation in PETCO_2 _should be expected [18,20,21]. This marked reduction in PETCO_2 _at low CO values might be attributed not only to limited CO_2 _elimination, but also to changes in metabolic CO_2 _production during oxygen supply dependency [21-23]. In contrast, at high CO values, pulmonary blood flow would not be the limiting factor for PETCO_2_, and thus, changes in PETCO_2 _would not reflect its variations but mainly the adequacy of alveolar ventilation [18,20]. Although our study was not designed to address this specific issue, the strong association observed during PLR between fractional changes in CO and PETCO_2 _suggests that, under the conditions described by the study protocol, this relationship remains valid over the wide range of studied CO values. Furthermore, the moderate magnitude of transient changes in CO produced by PLR (in contrast with marked and sustained decreases induced in some studies [18-21]) and the timing between PETCO_2 _and CO measurements [25] may also have contributed to the observed findings in our study. When perturbations in CO were produced over a brief period of time and PETCO_2 _measurements were performed within a relatively short time frame, similar results have been observed in previous studies [22,25].

Before drawing conclusions from our results, some considerations should be mentioned. First, the assessment of fluid responsiveness using PETCO_2 _variations should potentially share the same limitations as interpreting CO changes during PLR, since in this context the former is in essence just a surrogate for the latter. Therefore, any condition that could affect the ability of PLR to predict fluid responsiveness (for example, intraabdominal hypertension) [12] should also be considered when analyzing PETCO_2 _changes. Second, to prevent fluctuations in minute ventilation, all the patients were mechanically ventilated in volume control mode, deeply sedated and fully adapted to the ventilator with no inspiratory efforts. Consequently, in spontaneously breathing patients, the unpredictable variations in alveolar ventilation could make the analysis of changes in PETCO_2 _more difficult. For this reason, this population was deliberately excluded from the study and extrapolation of our results to this clinical condition cannot be recommended. Third, the assessment of PETCO_2 _variations is also based in the assumption that no significant changes in metabolic CO_2 _production have been produced while performing the PLR maneuver. If the metabolic rate varies considerably (for example, during shivering, fever, and so on), the observed changes in PETCO_2 _could not be attributed solely to modifications in CO and thus the ability of PETCO_2 _variations to predict fluid responsiveness could be diminished. However, the maximum hemodynamic effects of PLR take place primarily during the first minute [6], so it is very unlikely that this phenomenon will occur in such a short period of time. By contrast, since infusion time was significantly longer, this assumption is more difficult to sustain and could be one of the reasons for the weaker relationship between changes in CO and PETCO_2 _after fluid administration. However, even under these circumstances, the VE-induced increase in CO still accounted for more than 50% of the observed changes in PETCO_2_. Finally, the 5% cutoff found for changes in PETCO_2 _represents an increase of no more than 2 mmHg, which *a priori *could be attributed to a low signal-to-noise ratio and easily interpreted as a measurement error. Nonetheless, we effectively confirmed that this value is almost three times the LSC for PETCO_2 _measurement, so under the same conditions, an increase above this threshold should represent an actual increase in PETCO_2 _and the presence of fluid responsiveness. Despite these considerations, PETCO_2 _measurement could offer an easily available, totally noninvasive method for the assessment of fluid responsiveness with similar performance as measuring CO, but without the need of any hemodynamic monitoring device, when similar clinical conditions are present (uniform alveolar ventilation and constant VCO_2_).

## Conclusions

In our study we demonstrated that PETCO_2 _effectively tracked changes in CO during the PLR maneuver and predicted fluid responsiveness in patients with acute circulatory failure with fixed minute ventilation, assuming a constant tissue CO_2 _production.

## Abbreviations

ΔCO_PLR_: Passive leg raising-induced changes in cardiac output (%); ΔPETCO_2-PLR_: Passive leg raising-induced changes in partial end-tidal CO_2 _pressure (%); ΔPP_-PLR_: Passive leg raising-induced changes in arterial pulse pressure (%); Acc: Mean acceleration of aortic blood velocity; ARDS: Acute respiratory distress syndrome; CE: Coefficient of error; CI: Confidence interval; CO: Cardiac output; CV: Coefficient of variation; FTc: Corrected flow time; GRI: Guyatt's Responsiveness Index; IQR: Interquartile range; LSC: Least significant change; MAP: Mean arterial pressure; PEEP: Positive end-expiratory pressure; PETCO_2_: Partial end-tidal CO_2 _pressure; PLR: Passive leg raising; PP: Arterial pulse pressure; SV: Stroke volume; VCO_2_: Metabolic CO_2 _production; VE: Volume expansion.

## Competing interests

MIMG has received consulting fees from Edwards Lifesciences. The other authors have no competing interests to disclose.

## Authors' contributions

MIMG, AGC and JCDM conceived the study. MIMG designed the study, participated in the recruitment of patients, performed the statistical analysis, interpreted the data and drafted the manuscript. AGC participated in the study design, interpreted data and helped draft the manuscript. MGR, RMP and VPM participated in patient recruitment, data collection and technical support. All authors contributed to the critical review of the manuscript and approved the final manuscript.
